# A multi-query, multimodal, receiver-augmented solution to extract contemporary cardiology guideline information using large language models

**DOI:** 10.1093/ehjdh/ztaf111

**Published:** 2025-09-23

**Authors:** Robert M Radke, Gerhard-Paul Diller, Rohan G Reddy, Pushpa Shivaram, David A Danford, Shelby Kutty

**Affiliations:** Department of Cardiology III-Adult Congenital and Valvular Heart Disease, University Hospital Muenster, Muenster, Germany; Department of Cardiology III-Adult Congenital and Valvular Heart Disease, University Hospital Muenster, Muenster, Germany; Adult Congenital Heart Centre and National Centre for Pulmonary Hypertension, Royal Brompton and Harefield Hospitals, Guys & St Thomas’s NHS Trust, London, UK; National Heart & Lung Institute, Imperial College, London, UK; Department of Biology, The Johns Hopkins University, Baltimore, MD, USA; Division of Pediatric Cardiology, Department of Pediatrics, University of Augusta, Augusta, GA, USA; Academic Programs, BayCare Health System, 2985 Drew Street, Clearwater, FL 33759, USA; Academic Programs, BayCare Health System, 2985 Drew Street, Clearwater, FL 33759, USA

**Keywords:** Large language model, Retrieval-augmented generation, Clinical practice guidelines

## Abstract

**Aims:**

The aim of the current study was to assess the utility of a state-of-the-art large language model (LLM) based on curated, defined clinical practice recommendations to support clinicians in obtaining point-of-care guidelines for individual patient treatment while maintaining transparency.

**Methods and results:**

We combined cloud-based and locally run LLMs with versatile open-source tools to form a multi-query, multimodal, retrieval-augmented generation chain that closely reflects European cardiology guidelines in its responses. We compared the performance of this generation chain to other LLMs including GPT-3.5 and GPT-4 on a 306-question multiple-choice exam with questions consisting of short patient vignettes from various cardiology subspecialties, originally written to prepare candidates for the European Exam in Core Cardiology. On the multiple-choice test, our system demonstrated overall accuracy of 73.53%, while GPT-3.5 and GPT-4 had overall accuracies of 44.03 and 62.26%, respectively. Our system outperformed GPT-3.5 and GPT-4 for the following categories of questions: coronary artery disease, arrhythmia, other, valvular heart disease, cardiomyopathies, endocarditis, adult congenital heart disease, pericardial disease, cardio-oncology, pulmonary hypertension, and non-cardiac surgery. For maximum transparency, the system also provided reference quotes for its recommendations.

**Conclusion:**

Our system demonstrated superior performance in question-answering tasks on a set of core cardiology questions as compared with contemporary publicly available chat models. The current study illustrates that LLMs can be tailored to provide documented and accountable guideline recommendations towards actual clinical needs while ensuring recommendations are derived from up-to-date, trustable, and traceable documents.

## Graphical Abstract

**Graphical Abstract:**
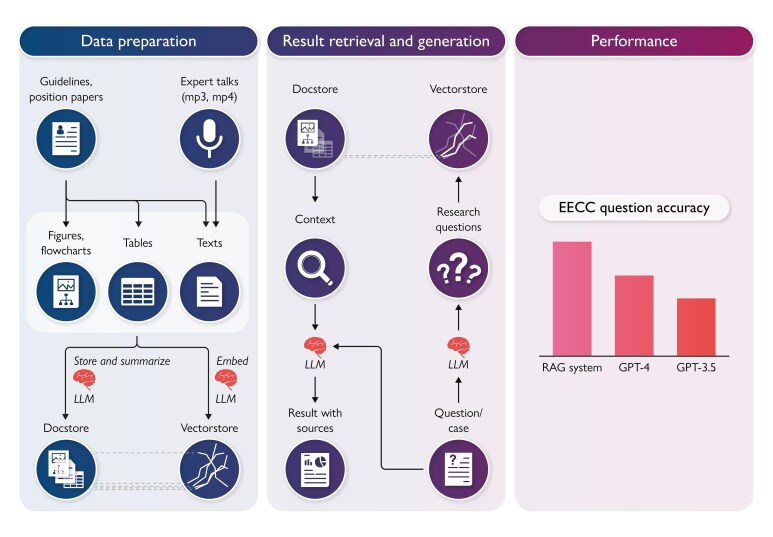

## Introduction

The European Society of Cardiology (ESC) guidelines are a central resource intended to optimize patient care by providing standardized, evidence-based assistance to cardiologists. Since its inception in 1950, the ESC has progressively mapped the field of cardiology and offered valuable resources to guide cardiologists in making informed patient care decisions.^1^ To facilitate cardiologists’ access to current recommendations, the ESC presents guidelines as PDF files, as a smartphone application, and through online webinars.^2^ However, the cardiology guidelines are frequently updated and refined, with as many as four to five guideline documents at each annual congress, accompanied by position papers and consensus statements. In the process, the guidelines have become more complex and detailed, and subject to important changes in keeping with advances in the field, making it increasingly difficult for clinicians to read and closely follow the recommendations in their entirety.^3,4^

One way to meet this challenge might be to take advantage of artificial intelligence (AI), in particular large language models (LLMs). These models are trained on vast amounts of heterogeneous text data from the open web and will generate natural language responses based on user input. It is not difficult to imagine how these might help clinicians efficiently and accurately extract case-specific advice from current clinical practice guidelines.

The potential for open-source software chains enhancing off-the-shelf LLMs to provide the best guideline-based answers has not yet been examined in depth. Therefore, we sought to explore how current cloud-based and locally run LLMs could be combined with a multi-query, multimodal, retrieval-augmented generation (RAG) chain that closely reflects European cardiology guidelines in its answers. The aim of this project was to demonstrate the feasibility of applying LLMs to a predefined, curated, and expert-maintained set of guideline recommendations to (i) provide advice focused only on current, relevant guidelines and (ii) achieve transparency by providing references to the applicable guidelines and the scientific foundation upon which the recommendations are based.

## Methods

### Hardware and software

Local code for data ingestion and inference was run on a Mac Studio M2 Ultra computer (Apple Inc.) with macOS 14.3.1. Code was written with Python 3.11.6 and using Langchain 0.2 library to utilize local and cloud-based LLMs, embedding models, and local data retrievers. OpenAI models GPT-3.5, GPT-4, and GPT-4-Vision-preview were used on the company’s servers. Models for embedding (BAAI/bge-large-en-v1.5) and summarization (Mixtral 8×7B, Mistral AI) were run locally with Ollama 0.1.28.^5^ ChromaDB,^6^ PostgreSQL,^7^ and elasticsearch (Elasticsearch B.V.) were used as local databases for ingested data. Conversion of source PDFs into text, graphics, and tables was partially automated with the Mathpix Pro service (Mathpix, Inc.). The web interface for the complete chain was created with gradio.app (Hugging Face, Inc.).

### Source data

The source data consisted of the latest set of publicly available official full guideline documents, which are under the licence of Oxford University Press, as of 2024 with texts, tables, flowcharts, images, and supplements, from the European Society of Cardiology (ESC) since 2015. Only the most recent guidelines for a given topic were included. Official position papers from the ESC working groups were added if deemed to be of sufficient general interest. To enhance understanding of diagnostic standards, official imaging recommendations for echocardiography and cardiovascular magnetic resonance (CMR) were included from the European Association of Cardiovascular Imaging (EACVI) and the Society for Cardiovascular Magnetic Resonance (SCMR). As proof of concept, a limited number of the latest additional guideline-themed podcast materials from the ESC were also incorporated.

### Data preprocessing

**Figure 1:**
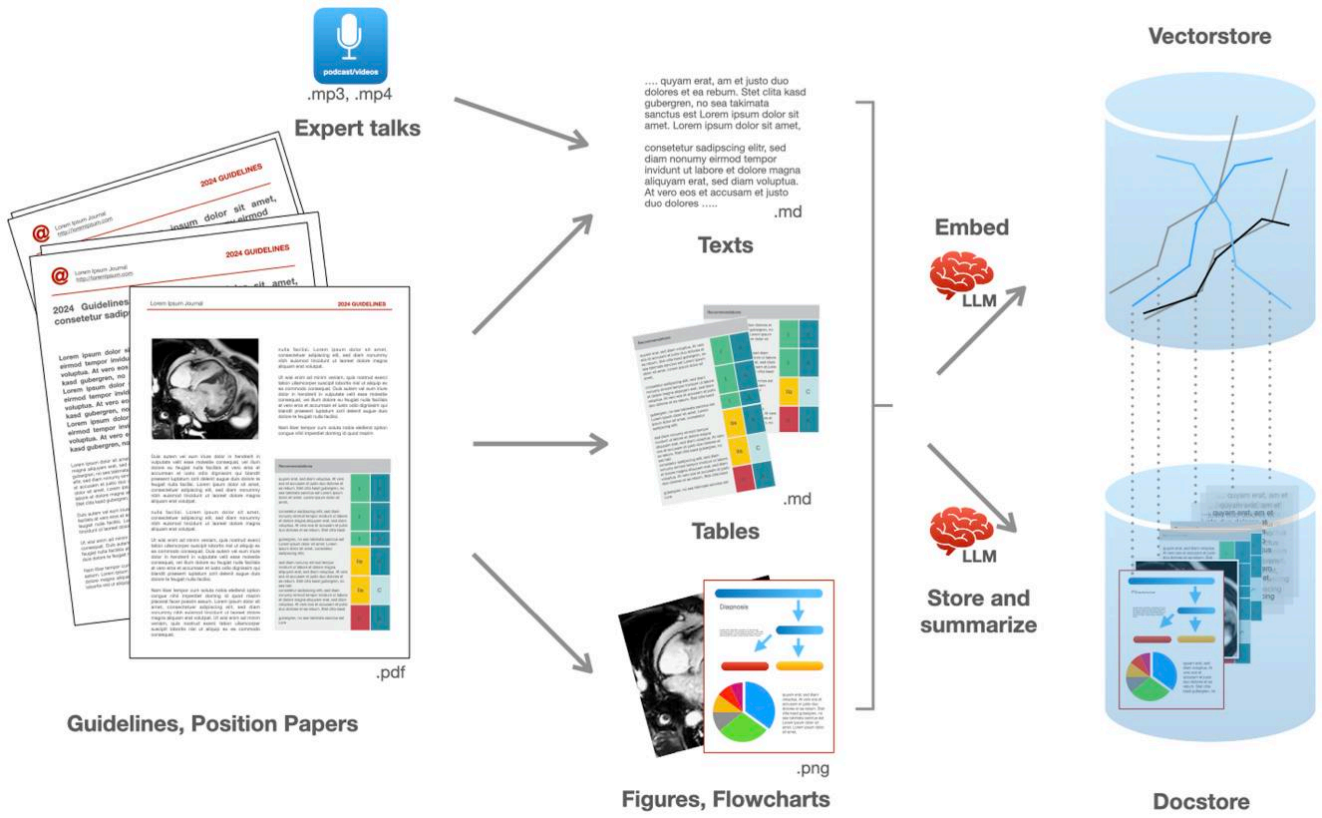
Components of and data preparation for multimodal retrieval-augmented generation. Plain text information, tables, and figures are extracted from pdf documents through a only partly automated process to ensure exact representation. Data are split into parts (‘chunking’). During ‘embedding,’ content and meaning are converted into a number-based representation, which is saved into a vector-database (vectorstore). The original data ‘chunks’ are stored in a document database (docstore) for later retrieval. Vector representation and original data are linked with unique document IDs. Contents such as guidelines or podcasts can be added at any time.

The raw extracted text from the PDF documents was preprocessed to remove references and unwanted links. Tables and images, along with their legends, were extracted from their positions in the text and processed separately. Chapter headings and subheadings were categorized, marked, and served as metadata to conserve context. The cleaned texts were split into smaller chunks by headings and further subdivided if they were still too large for efficient retrieval. Text chunks were sent to an embedding model to generate vector representations, which were then stored in the vector database for later semantic search. Each chunk of text itself was linked and stored in the document database. Tables were processed similarly but to assure complete retrieval they were not split into chunks. To enhance retrievability, local LLMs were employed to generate short summaries and hypothetical questions for each text chunk, which were also embedded and stored in the vector store. Thus, multiple vector entries could point to a single chunk in the data storage (multi-vector retriever) (*Figure 1*).

### Image and audio processing

Images extracted from the PDFs were manually checked for accuracy and combined with their legends, titles, headings, and subheadings. Flowcharts, images, and legends were passed to GPT-4-Vision to generate textual descriptions, which were then embedded in the vector database and linked to the original image files stored in the document database. Finally, official guideline-themed audio content was passed to an OpenAI speech-to-text endpoint and converted to plain text documents. These text documents were processed in the same manner as described above (*Figure 1*).

### Data retrieval

**Figure 2:**
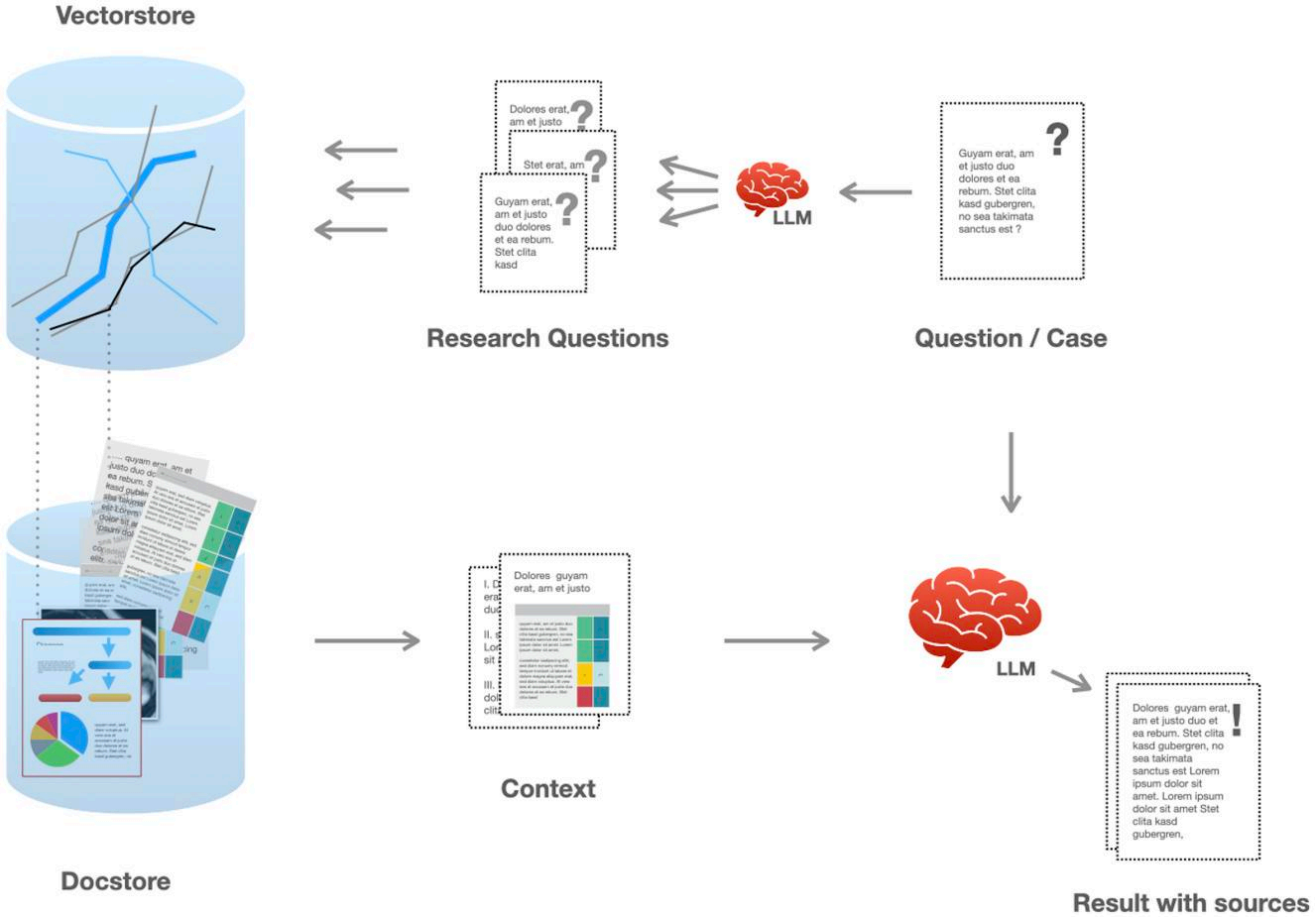
Retrieval of multimodal context and result generation. User input in the form of a specific question or a clinical case scenario is passed into a predefined chain of functions and LLM models. First, a model is used to generate further ‘research questions’ to optimize information retrieval. Then, the questions are converted to a number representation (embedding) based on their meaning. Searches in the vector-database will return vectors semantically similar to the questions, so that any helpful chunks from the document database can be retrieved. Finally, the gathered context is presented to the LLM together with the original user question for result generation. The unaltered context is available to the user for result verification.

The LLM chain is optimized for two different scenarios: an interactive chat mode and a multiple-choice answering mode. In chat mode, the user’s question is passed into a preprocessing chain to generate additional questions related to the material for retrieval.^8^ These questions are then embedded into vector representations and passed to retriever functions, which search the vector database using semantic similarity and return several entries. The corresponding texts, tables, images, or audio transcriptions are retrieved from the document storage. In addition, specific text items like disease entities, medications, or highly specific medical terms are extracted by a local LLM and passed to a keyword search to augment the similarity results. The initial list of contents is then processed by a ranking algorithm where each element is scored based on its usefulness concerning the original user input.^9,10^ Only the top 10 entries make it into the context list for the particular question, resulting in a highly relevant multimodal collection of relevant guideline content (*Figure 2*). The quality of the vector database and RAG-information retrieval system was evaluated using manual pilot queries across topics. Contextual outputs were cross-checked with the original ESC guideline content to verify that key recommendations, flowcharts, and definitions were correctly retrieved and grounded.

### Model selection

For high-quantity and low-risk tasks state of the art models are used. For difficult tasks like question answering based on context and user input, the latest state-of-the-art models are employed due to their larger number of parameters, faster performance, and ability to work with larger context windows with less degradation.^11^ The ‘temperature’ parameter is set to 0 to make the output more predictable and repeatable.

### Prompting

We employ several common enhancement techniques in our prompts. The model is asked to adopt a specific persona to set the tone for the kind of answers we want to receive. We include the retrieved context and explain how to interpret it, providing a few examples through ‘few-shot’ prompting. We instruct the model to answer based solely on the provided context and cite the source. Newer information is to be preferred over older information, as newer guidelines may override recommendations from older, thematically overlapping guidelines. In the context documents, we employ Markdown to structure headers and text hierarchically.^12^ In multiple-choice question mode, we ask for a structured stepwise approach similar to SOAP notes in clinical practice. The primary prompt measured ∼1200 tokens, and the total context length used, which includes both the input (prompt + retrieved documents) and the output, was kept well below the model’s maximum of 128 000 tokens for GPT-4 Vision and GPT-4o. Including too few retrieved items led to incomplete answers, while including too many items led to quality degradation where important context items in the middle of the document could be overlooked.

### Generating an answer

The context and prompt are combined into a message and passed to the LLM. In the chat mode, a familiar chat interface allows users to type or paste their questions. While answers are streamed as they are generated (as users are accustomed to), the process of context retrieval and ranking takes some time. The output text provides structured answers and cites the source document it is based on as well and mentions relevant guideline images. Five local cardiologists (two focused on valvular heart disease/congenital heart disease, two focused on coronary artery disease, and one focused on arrhythmia) were included in testing the quality of free-form dialogue answers to optimize the retrieval parameters and the prompts. To provide a transparent result, the original and unaltered retrieved images, tables, and texts are presented on additional pages.

### Multiple-choice evaluation

To evaluate the performance of our RAG system, a set of non-public, commercially available preparation questions of fixed category distributions used by candidates for the European Exam in Core Cardiology (EECC) was acquired (StudyPRN, Learna Ltd). These questions were selected due to their use in earlier studies on LLMs in the European context and their commercial nature, which may reduce the likelihood of their inclusion in the LLM’s training data.^13^ Questions containing audio or visual assets were excluded. The 306 text questions consist of short patient vignettes from various cardiology subspecialties (*Table 1*). Each question included a designated ‘correct’ answer, an explanation, and references to the appropriate guidelines. To optimize the system’s performance on the question set, the prompt was written to provide basic instructions and recommendations for answering multiple-choice questions. These instructions included reading and structuring the available information, reviewing all answer options before making a selection, and responding with only the letter corresponding to the chosen answer. The input of questions into the system, as well as the extraction of answer choices from the system, was fully automated. The question–answer pairs were stored in a database. Input of questions into the system and extraction of answers was done through a scripted pipeline. In our comparative analysis, we evaluated the performance of two commonly used and unaltered language models, GPT-3.5 and GPT-4 (OpenAI), powering ‘ChatGPT’ with the same set of questions.

**Table 1:** Overview of topics covered by the cardiology multiple-choice text question used

Category	Number	Percent
CAD	64	21%
Arrhythmia	54	18%
Other	41	13%
Valvular heart disease	35	11%
Heart failure	22	7%
Cardiomyopathies	18	6%
Endocarditis	14	5%
ACHD	14	5%
Sports	9	3%
Pericardial disease	8	3%
Pregnancy	8	3%
Cardio-oncology	7	2%
Pulmonary Hypertension	7	2%
Non-cardiac surgery	5	2%
Total	306	

306 commercially available multiple-choice text questions aimed at preparing candidates for the European Exam in Core Cardiology (EECC) were used to evaluate model performances. Questions and provided answers were based on ESC guidelines.

## Results

### Multiple-choice evaluation

The 306 multiple-choice test questions were categorized into 14 topics, but more than half of the questions focused on 4 of these: coronary artery disease (21%), arrhythmia (18%), valvular heart disease (11%), and heart failure (7%). See *Table 1* for detailed representation of questions across all 14 topics.

The RAG system demonstrated an overall accuracy of 73.53%, outperforming GPT-3.5 and GPT-4, which had overall accuracies of 44.03 and 62.26%, respectively. The accuracy of our model chain was superior to GPT-3.5 and GPT-4 in 11 of the categories: CAD, arrhythmia, other, VHD, cardiomyopathies, endocarditis, ACHD, pericardial disease, cardio-oncology, pulmonary hypertension, and non-cardiac surgery. The performance of the system was particularly notable in the topics of cardiomyopathies and endocarditis, achieving accuracy of 85.71% in both categories. It reached a perfect accuracy rate of 100% in cardio-oncology, far surpassing both GPT-3.5 and GPT-4, which scored 42.86 and 71.43%, respectively.

The RAG system’s performance was on par with GPT-4 in the heart failure and pregnancy categories, sharing accuracy rates of 63.64 and 87.5%, respectively. In the Sports Cardiology category, the RAG system’s accuracy was below GPT-4 (77.78% vs. 88.89%). Comprehensive accuracy rates for each question category are displayed in *Table 2*.

**Table 2:** Performance of our multi-query, multimodal, receiver augmented generation chain in answering 306 multiple-choice cardiology text questions

	GPT-3.5	GPT-4	RAG System
CAD	43.75%	60.94%	67.19%
Arrhythmia	33.93%	46.43%	62.96%
Other	52.38%	71.43%	80.49%
Valvular heart disease	47.22%	61.11%	77.14%
Heart failure	45.45%	63.64%	63.64%
Cardiomyopathies	50.00%	79.17%	85.71%
Endocarditis	35.71%	57.14%	85.71%
ACHD	42.86%	57.14%	85.71%
Sports	77.78%	88.89%	77.78%
Pericardial disease	25.00%	50.00%	62.50%
Pregnancy	50.00%	87.50%	87.50%
Cardio-oncology	42.86%	71.43%	100.00%
Pulmonary hypertension	25.00%	62.50%	71.43%
Non-cardiac surgery	50.00%	50.00%	60.00%
Total	44.03%	62.26%	73.53%

Results in different topic categories and total results compared with results from generally available models are shown.

## Discussion

### The RAG system as a clinical tool

Front-line clinicians who obtain recommendations from web-based LLM tools such as ChatGPT may be poorly served, so improved LLM chains are necessary. Thus, we sought to evaluate a multi-query, multimodal RAG solution to extract contemporary cardiology guideline information using LLMs. Our RAG system demonstrated superior overall accuracy on a multiple-choice question test aimed to prepare candidates for the EECC when compared with GPT-3.5 and GPT-4.

Previous studies have shown that GPT models including GPT-3.5 and GPT-4 are able to answer medical questions including USMLE style questions at a passing rate.^14^ Specific to cardiology, Milutinovic *et al*. evaluated the performance of ChatGPT (versions 3.5 and 4.0) in answering multiple-choice cardiovascular medicine questions from the Medical Knowledge Self-Assessment Program (MKSAP). While GPT-4 exhibited an accuracy rate of 74.5%, which outperformed internal medicine residents and physicians, this was inferior to the 85.7% accuracy rate seen from cardiology attending physicians.^15^ Moreover, Malkani *et al*. found that GPT-4 had an accuracy rate of 87% on cardiovascular questions from MKSAP 19, which was higher than the 60% accuracy averaged among all human users. However, despite GPT-4’s high accuracy rate, it is worth noting that all of the questions that were answered incorrectly were related to clinical management and treatment, an area that is of high importance if LLMs like GPT-4 are to be used as a support tool at the point of care.^16^ Additionally, the performance of LLMs will likely need to exceed or be on par with attending cardiologists for its utilization as a clinical decision support tool, highlighting the need for improved LLMs. In this context, our RAG system, which demonstrated an overall accuracy rate of 73.53%, outperforming both GPT-4 and GPT-3.5, may serve as a promising clinical decision support tool at the point of care.

Adding to the strengths of our system, it also limits inaccuracies such as ‘hallucinations’, which may be due to misleading information in the source data or simply the model generating text based on what it assumes is expected, by combining a vector knowledge base with prompting techniques and newer models with vision capabilities.^17,18^ While current LLMs primarily store knowledge within their parametric memory and are inherently limited by being trained on a dataset containing information up to a certain knowledge cutoff date, our use of RAG integrates external, verifiable knowledge sources to provide up-to-date and contextually grounded information.^19^ Additionally, while training medical-focused LLMs is a logical approach, it requires extensive resources.^20,21^ In contrast, effective prompting strategies may be a resource efficient alternative solution.^22^ Our RAG system reduces the need for fine-tuning and results in a flexible model that can align to a single source of truth while using much less labelled data and computational resources.

Another major strength of our system is its accessibility and transparency. According to Rajpurkar *et al.*, medical AI systems gain user trust, through transparent provision of references upon which recommendations are based. Unfortunately, many LLMs are ‘black boxes’, which cannot interpret with certainty how the system came to its conclusions, thus limiting their applicability to clinical practice.^23^ In chat mode, our model chain is able to address simple questions about guideline contents and generate structured and logical answers based on and citing the latest ESC guidelines. Context, including recommendation tables, flowcharts, and text details, is drawn from all relevant guideline documents and is presented for user review in the ‘Sources’ tab in the user interface, thereby improving the accessibility of information.

Generic LLMs are trained with source data that often do not contain many medical documents, limiting the quality and depth of recommendations and solutions to specialized medical queries.^24^ In contrast, the source data for our system are the latest set of publicly available official full ESC documents, including supplements, since 2015, which can also be updated within minutes to provide more clinically relevant recommendations.

### Limitations and future direction

Our study, while demonstrating the potential of utilizing a model chain integrating RAG and advanced prompting techniques, has important limitations. The RAG system, which is not yet publicly available, did not completely prevent the provision of incorrect information due to the inherent limitations of current LLMs in distinguishing between factual content and plausible but incorrect information. It also suffers from occasional misunderstandings of clinical vignettes due in part to a lack of specificity of medical terminology. Often, terms were too specialized for the embedding model even when we employed a hybrid search to mitigate this issue. A more finely tuned medical embedding model could significantly enhance performance by better distinguishing between disease entities, medications, and other specific terms.

The calculation of medical scores presents another challenge. Current LLMs may struggle with seemingly simple computational tasks, such as score calculations found in guidelines. Integrating specialized tools or agents to perform deterministic calculations could improve the system. Moreover, the absence of an official benchmark test posed a challenge to our evaluation of the RAG system. The questions used were intended for test preparation and have been used in prior work, but they may or may not be sufficient to evaluate a model’s performance in a clinically useful way. While most questions and references were detailed and up-to-date, we identified discrepancies where the system’s ‘incorrect’ answers were actually in line with the most recent guidelines, revealing that some of the test material was outdated. This discrepancy, particularly evident in questions related to endocarditis based on the 2015 guidelines, highlights the importance of continuously updating the test material to reflect the latest scientific consensus. Moreover, while we included only the most recent guidelines on a given topic in our system, even up-to-date guidelines may overlap in certain areas. In cases where there is partial overlap between guidelines, both are retained. Additionally, visual and audio questions were not analysed as the analysis of audio and clinical images, such as echocardiographic or magnetic resonance images, is limited, model-dependent, and cannot be augmented enough by a RAG-type system.

The limitations and challenges identified in our study point to several avenues for future research and development. Hallucinations and the reliance on implicit knowledge are shortcomings that require further refinement of the system’s processing and interpretation in context. Enhancing the system’s medical terminology literacy through more specialized embedding models could also improve accuracy and reliability. The integration of deterministic calculation tools represents another promising direction, potentially expanding the system’s utility in clinical decision-making. Additionally, the use of the PDF format for clinical practice guidelines poses significant challenges for accurate data ingestion and interpretation by AI systems. The complex layout of PDFs, with images, tables, and other visual elements, can disrupt the continuity of the text and lead to errors in the extracted information, which were corrected manually. As AI increasingly penetrates clinical medicine, it makes sense for future guidelines to be drafted in more machine-readable formats especially for decision trees to facilitate more accurate data assimilation and interpretation by AI models, thereby reducing errors. Furthermore, as the performance of LLMs on questions containing clinical images or audio remains limited, it is important that future LLMs have robust image and audio analysis capabilities to assist in image and audio based clinical decision-making.

Moreover, privacy remains a paramount concern, especially in the context of potentially identifiable patient data. The use of local models emerges as a viable alternative to address GDPR and other privacy regulations, offering a pathway to leverage LLMs’ capabilities while safeguarding patient information. Lastly, with the increasing popularity of medically trained LLMs like OpenEvidence, future areas of research may include the utilization of OpenEvidence to validate our RAG system, particularly as LLMs evolve to include guideline-based evidence.

## Conclusion

We implemented a complex semantic database system with good retrieval of relevant multimodal documents across the whole spectrum of the latest guidelines in cardiology. Leveraging versatile open-source tools with local and cloud-based LLMs, we demonstrated superior performance in question-answering tasks on a set of core cardiology questions compared with publicly available chat models. RAG and advanced prompting techniques, despite their limitations, hold promise for improving the factual accuracy and utility of LLMs in medical contexts. As LLMs continue to evolve, the incorporation of these techniques, along with ongoing refinement and adaptation to the specific challenges of medical information processing, will be crucial in harnessing the full potential of AI in healthcare.

## Data Availability

The RAG system developed in this study is not publicly available due to copyright restrictions on ESC guideline documents. The underlying guideline data are accessible through the European Society of Cardiology (https://www.escardio.org/Guidelines/Clinical-Practice-Guidelines). Processed data and code may be shared upon reasonable request to the corresponding author.
